# Co-Designing a Social Connections Program With Older Adults

**DOI:** 10.1093/geroni/igab046.1027

**Published:** 2021-12-17

**Authors:** Jasmine Chandy, Padraic Stanley, Grisel Rodriguez-Morales, Erin Emery-Tiburcio

**Affiliations:** 1 Rush University Medical Center, Chicago, Illinois, United States; 2 Rush University, Chicago, Illinois, United States

## Abstract

Loneliness presents a higher risk for mortality than smoking 15 cigarettes per day. COVID-19 has exacerbated loneliness for many older adults, without access to family, friends, and community. Friendly caller programs utilizing volunteers to talk with older adults who are lonely can be helpful, providing much-needed contact. However, few lasting connections have formed in these programs. To enhance our social connections program, we systematically engaged a group of older adults who struggle with social isolation to co-design a program to meet their needs. This group met virtually twice for two hours to (1) identify contributors to their isolation, generate ideas for ideal program components, and how best to connect older adults to each other; and (2) to refine the multi-component program created by staff based on the first discussion. Group process and themes will be presented, along with a discussion of key issues in program co-design with older adults.

